# Simulation Analysis of the Chemical Mechanical Polishing Process for Monocrystal 4H-Silicon Carbide Based on Molecular Dynamics

**DOI:** 10.3390/mi16121350

**Published:** 2025-11-28

**Authors:** Yang Lei, Weigang Guo, Kaiping Feng, Zitong Sun

**Affiliations:** 1 Special Equipment Institute, Hangzhou Polytechnic University, No. 68, Xueyuan Street, Hangzhou 310018, China; leiyangcn@126.com (Y.L.);; 2 College of Mechanical Engineering, Quzhou University, No.78, North Jiuhua Road, Quzhou 324000, China

**Keywords:** silicon carbide, chemical mechanical polishing, molecular dynamics, polishing depth, polishing speed

## Abstract

This paper delves into the mechanism of the chemical and mechanical action during chemical mechanical polishing (CMP) of monocrystal silicon carbide (SiC) through the molecular dynamics (MD) method. The oxidation simulation showed that the Si atoms mainly reacted in the form of Si-O and Si-H, while the C atoms are in the form of C-O. The impact of the sliding depth and the polishing speed on the SiC workpiece was analyzed. Results show that more substrate atoms are removed as the polishing depth and speed increase. When the polishing depth reached 8 angstroms, 624 atoms were removed from the substrate. At the same time, the increased diamond polishing speed expands the polishing area. This reduces the indentation of the cut atoms on the surface of the workpiece and increases the removal efficiency of the SiC surface atoms, and when the polishing speed reached 125 m/s, the instantaneous temperature reached about 800 °C. In short, the polishing depth and speed have a significant impact on the polishing process, and the polishing depth has a more sophisticated influence on the atom removal rate.

## 1. Introduction

Semiconductor materials are the core building blocks of the rapid development of information technology, which has important implications for national strategies [1]. However, faced with the challenges of special scenarios and extreme environments, the original dominant silicon (Si) and the subsequent development of gallium arsenide (GaAs) show their inherent limitations and have been difficult to meet the growing demand. There was an urgent need for an electronic device that could work reliably in extreme environments [2]. As a result, SiC, the third-generation semiconductor material, is gradually becoming widely used in various industries due to its superior performance advantages.

SiC has excellent physical and chemical properties such as high hardness, high melting point, high thermal conductivity, and high chemical stability [3]. This makes it the most suitable substrate material for power electronic devices. This makes semiconductor materials represented by SiC widely used as a new generation of substrate materials for power electronic devices. As a substrate material for the manufacture of high-performance microelectronics and optoelectronic devices, SiC not only ensures high surface accuracy and sub-nanometer roughness during processing but also avoids surface and sub-surface damage [4]. However, as SiC is a typical hard and brittle material, its processing faces problems such as low efficiency [5], high cost [6], and being unfriendly to the environment [7,8].

**Figure 1:**
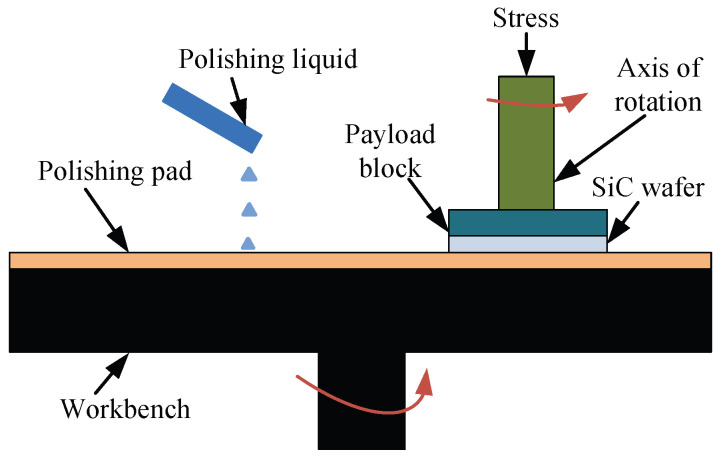
Schematic diagram of chemical mechanical polishing.

In order to obtain the SiC substrate that satisfies the requirements, researchers have developed a variety of precision grinding and polishing processing technologies, such as mechanical polishing, plasma polishing, and CMP [9]. This paper mainly studies the technology of CMP. It is a polishing method that combines chemical and mechanical action. First, a softening film is generated on the surface of the workpiece through chemical action, and then the film is removed by mechanical action, as shown in Figure 1. In 1997, Zhou L et al. first proposed the chemical mechanical polishing test of SiC [10]. The surface roughness of SiC is greatly reduced by controlling the temperature, acidity, and alkalinity of the solution. Subsequently, in order to improve the surface polishing quality of SiC, researchers focused on the impact of parameters such as polishing fluid, polishing head, polishing pressure, and temperature on the surface polishing effect [11]. For example, Lee et al. added chemical reagents to the polishing liquid to polish SiC. The results show that the addition of chemical reagents can improve the quality of the SiC surface and can effectively remove surface scratches [12]. Chen et al. studied the effect of colloidal SiO_2_ particles on the chemical mechanical polishing of 6H-SiC [13]. Pang et al. studied the roughness of SiC wafer polishing the Si surface under different polishing pressures and found that the surface roughness of the SiC wafer after polishing will deteriorate with the increase in polishing pressure [14]. Xue et al. studied the uniformity of material removal on the wafer surface at different relative speeds in dry tribological chemical mechanical polishing (DTCMP). The experimental results show that with the increase in rotational speed, the surface MRR of the chip increases first and then decreases [15]. However, almost all of the above studies focus on the macro scale, and the mechanism of material removal is still unclear and needs to be studied.

The chemical and mechanical action during CMP is nano or even sub-nano and occurs in femtoseconds, which makes the detection of the CMP process difficult. However, the MD method provides an insight into the sub-nanoscale and femtosecond time scale, which makes the observation of chemical and mechanical action possible. For example, Zhai et al. established an atomic model of SiC using molecular dynamics methods to study the material removal mechanism at the atomic level during the chemical mechanical polishing of cubic SiC under ultrasonic vibration [16]. The results show that the SiC workpiece has a local amorphous change in the process of machining. The introduction of ultrasonic vibration will greatly reduce the average tangential force and average normal force on abrasive particles, which is conducive to the progress of scratching and the improvement of surface quality. Wang et al. established a model of polishing a SiC workpiece with diamond abrasive particles based on molecular dynamics to study the effect of polishing pressure on the material surface [17]. Sun et al. [18] used the reaction force field (ReaxFF) molecular dynamics method to simulate the surface microscopic behavior of SiC during CMP with diamond abrasive and silica abrasive, respectively, to explore the removal mechanism of atoms on the surface of SiC with different hardness abrasives. The results show that silica abrasives are more susceptible to chemical reaction than diamond abrasives during polishing [18]. Diamond abrasive particles can enable more atoms to be removed from the surface of SiC, and silica can ensure better surface quality. Tang et al. [19] studied the effect of abrasive particle vibration on the microstructure evolution and material removal of SiC CMP. The results show that the particle vibration is beneficial to increase the atomic confusion on the surface of the workpiece and promote the SiC to participate in the oxidation reaction to form an oxide layer, which is convenient for mechanical removal [19]. Wang et al. used reactive molecular dynamics simulations to investigate the atom removal mechanism of the CMP process on the surface of Si (110) [20]. Ban et al. used a molecular dynamics approach to investigate the effects of different amplitudes and frequencies on the grinding effect of single-crystal diamond in 4H-SiC under ultrasonic and non-ultrasonic vibrations [21]. Gao et al. systematically investigated the deformation and crack formation in single-grain grinding of single-crystal 4H-SiC [22]. He et al. used the ReaxFF reactive force field to simulate CMP of SiC to reveal the mechanism of oxidation and removal of Si/C atoms in the polishing process of hydroxyl radical aqueous solution [23]. Wang et al. explored the anisotropy of 4H-SiC during the oxidation process; results show that accelerating the oxidation efficiency of the Si-Face is crucial in the double-sided CMP process [24]. Kang et al. investigated the effect of the Fenton reagent on the polishing behaviors of the SiC wafer and revealed the corresponding polishing mechanism [25]. He et al. considered the effect of alcoholic additives on polishing performance [26]. Cui et al. explore the influence of different oxidants on the polishing performance [27]. Zhao et al. employed the photoelectrochemical method to enhance the polishing process of SiC; results show that when the voltage reached 5 V, a damage-free surface can be obtained (S_a_ = 0.13 nm) [28]. Han et al. investigated the effect of the Fenton reaction on the polishing process, and after polishing, a minimum roughness value of R_a_ 0.78 nm can be obtained [29].

Though the MD simulation about SiC is quite comprehensive, present research mainly focuses on the reaction process of abrasives, while the oxidation potential of polishing fluid remains unexplored. In addition, the above-mentioned studies have generally focused on the ultrasonic vibration of SiC during machining, as well as the effects of abrasive and polishing pressure. The mechanism of material removal in chemical mechanical polishing of single-crystal 4H-SiC in the experiment is less studied at the atomic scale. In this paper, the MD model of single-crystal 4H-SiC nano-polishing was established to study the effect of polishing parameters on the machining process. Through the analysis of surface morphology, micro-crack formation mechanism, subsurface grinding force, and temperature, the material removal mechanism of single-crystal 4H-SiC in nano-polishing and the influence mechanism of the polishing process on it were revealed.

## 2. Simulation Method

### 2.1. Experiment Preparation

Before simulation, a CMP model of SiC built with the LAMMPS software (version: 2024.June 27) can simulate the motion of SiC atoms during the actual polishing process. The sliding simulation was performed at the (0001) crystal plane (Si face). The SiC matrix in the model is divided into a fixed layer, a constant temperature layer, and a Newton layer. The fixed layer remains stationary, which ensures that the position of the workpiece does not change during the simulation. The temperature of the thermostatic layer is set to 300 K, which balances the temperature of the entire simulation system and absorbs the heat generated by the Newton layer due to friction and other factors. In the simulation process, periodic boundary conditions are adopted in the X, Y, and Z directions to reduce the boundary effect in the simulation process. The SiC CMP model was simulated for 30,000 steps with a step size of 0.1 fs per step to ensure the accuracy and detail of the simulation.

### 2.2. Chemical Reaction Model

Direct observation of atomic bonding and bond breaking is extremely difficult in chemical reactions. Therefore, it is necessary to use a computer to construct chemical reaction models of SiC polishing in order to deeply explore the chemical bond relations among its internal atoms. The main focus of the model is the chemical reaction between the SiC workpiece and the polishing liquid. As a result, the model includes only two parts: the polishing liquid and the SiC workpiece.

**Figure 2:**
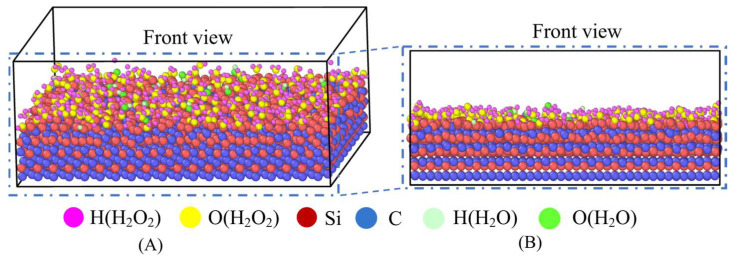
Polishing model of SiC and 30 wt% H_2_O_2_ solution. (**A**) Three-dimensional diagram; (**B**) main view.

LAMMPS software was used to construct the polishing model for SiC and a 30 wt% H_2_O_2_ solution. Its 3D plot and main view are shown in Figure 2. The core of the model is to show the interaction between SiC and H_2_O_2_ molecules in the form of 4H-SiC stacks. In this model, the bottom SiC layer is set as a fixed layer, which is designed to ensure that the SiC workpiece remains stable during the chemical reaction and prevents the atoms from moving. The remaining part reacts with the polishing liquid as a reaction layer. The polishing liquid is a 30 wt% H_2_O_2_ solution whose molecules interact with the SiC surface to simulate the reaction of interatomic bonds during the actual polishing process. The size of the model box is set to 75 Å × 54 Å × 25 Å. Parameters for chemical reaction simulation are shown in Table 1.

### 2.3. CMP Model

In the process of CMP of SiC, it is also difficult to directly observe the atomic motion under microscopic conditions. Therefore, a CMP model of SiC was constructed to investigate the mechanism of atom removal in the polishing process. This model focuses on the CMP of SiC slid by diamond particles. Thus, the model consists of three parts: abrasive particles, polishing fluid, and a SiC substrate. In this case, a spherical diamond was chosen as the abrasive particle, and its radius was set to 8 Å. The size of the simulation box is 70 Å × 40 Å × 66 Å.

**Figure 3:**
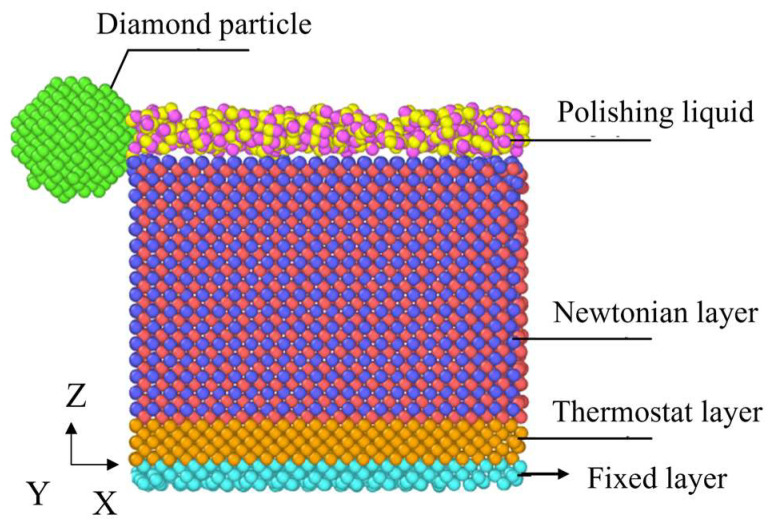
Model of CMP of SiC single crystal.

In the model, the SiC substrate is divided into three levels, from top to bottom, according to its functional properties: Newtonian layer, constant temperature layer, and fixed layer. A CMP model of a single crystal of SiC is shown in Figure 3. The Newtonian layer at the top of the model is 33 Å thick. This is the main region of the simulation process and can directly reflect the interatomic interactions in the polishing process. The brownish-yellow color in the middle of the model is a constant temperature layer with a thickness of 5 Å, whose temperature is set to 300 K. The lowest pinned layer is also 5 Å thick. The simulation parameters for CMP are given in Table 2. The interaction between diamond, polishing liquid, and SiC substrate was described by the Lennard-Jones potential, and their corresponding parameters are shown in Table 3. The calculation of the LJ potential is conducted through Equations (1) and (2). During the simulation, the epsilon and sigma values of the H atom are considered as 0 in the LJ potential; thus, the epsilon and sigma values of Si-H. C_SiC_-H, C_diamond_-H is considered as 0.

(1) $$\varepsilon_{ij}=\sqrt{\varepsilon_{i}*\varepsilon_{j}}$$

(2) $$\sigma_{ij}=\sqrt{\sigma_{i}*\sigma_{j}}$$

### 2.4. Analytical and Modeling Method

The simulation process is divided into the following stages:

(1) Build a suitable model system.
(2) By delaying the construction model system, the energy of the system is minimized, and the molecular potential energy is lowest.
(3) A chemical reaction occurs between SiC and a hydroxyl radical aqueous solution.
(4) Diamond abrasive particles move along the X-axis direction at different speeds and depths.
(5) Analyze the data of the polishing process.

**Figure 4:**
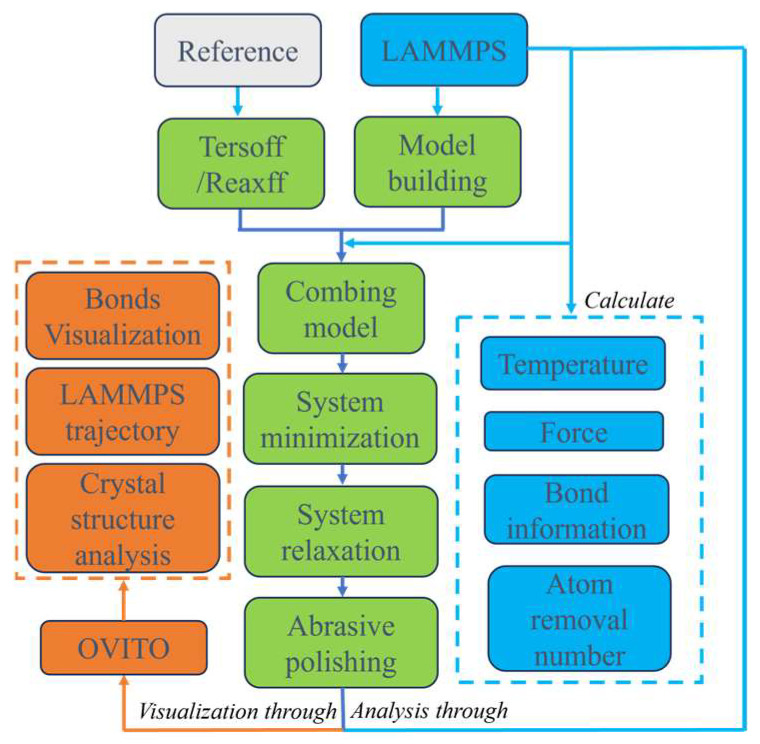
Analytic schematic after MD.

**Figure 5:**
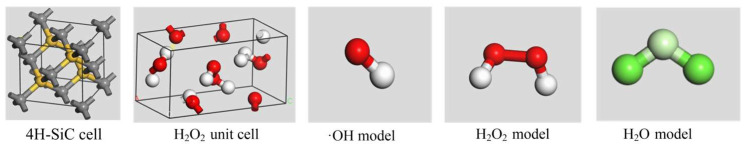
Model of each molecular group.

The main MD flowchart and analytic schematic are shown in Figure 4, and the theoretical bonding between the H_2_O_2_ polishing solution and the surface of the SiC workpiece is shown in Figure 5.

Due to the non-visual nature of LAMMPS, the simulation results can be displayed using OVITO software (version: basic 3.14.1). In this study, the diamond structure identification method was used to observe the change in crystal structure after polishing. At the same time, the surface morphology of the workpiece after polishing is observed by color coding, and the number of atoms above the workpiece is counted to analyze the polishing effect of SiC. In addition, in order to further understand the removal mechanism of the material atoms, temperature and force variation during the sliding process were calculated through the compute command embedded in the LAMMPS. The polishing velocity employed in this study was set within the range of 50–125 m/s. It is important to note that this value is significantly higher than typical experimental conditions. This choice is a well-considered compromise mandated by the computational constraints of MD. The MD method operates on a femtosecond timescale, making it computationally intractable to simulate the entire polishing process at experimental strain rates within a reasonable timeframe. The selected high velocity ensures that the critical atomic-scale events of interest—such as dislocation, plastic flow, and chip formation—can be observed within a feasible simulation duration. While this approach induces high strain rates and associated transient effects like localized adiabatic heating, it is a standard and established practice within the field of nanoscale tribological simulations. Its primary utility lies in enabling the qualitative investigation of fundamental deformation mechanisms, which is the central objective of this work, rather than the quantitative replication of experimental data.

## 3. Simulation Results

### 3.1. Experimental Results of Chemical Reactions

**Figure 6:**
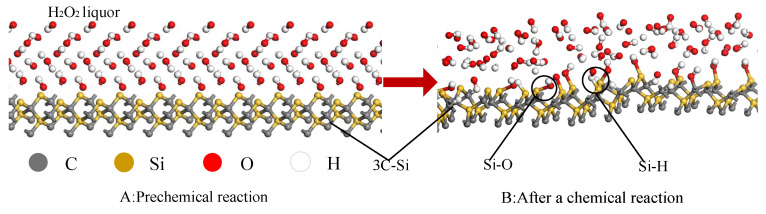
Theoretical bonding diagram of chemical reactions.

All the cells and molecules used in the chemical reaction simulation are depicted in Figure 5, including the 4H-SiC single-crystal cell, H_2_O_2_ cell, OH group, H_2_O_2_ molecule, and water molecule. During the CMP process, the polishing liquid of H_2_O_2_ will chemically react with the surface of the SiC workpiece and accompany the formation and fracture of bonds, thus promoting polishing efficiency, as shown in Figure 6A.

**Figure 7:**
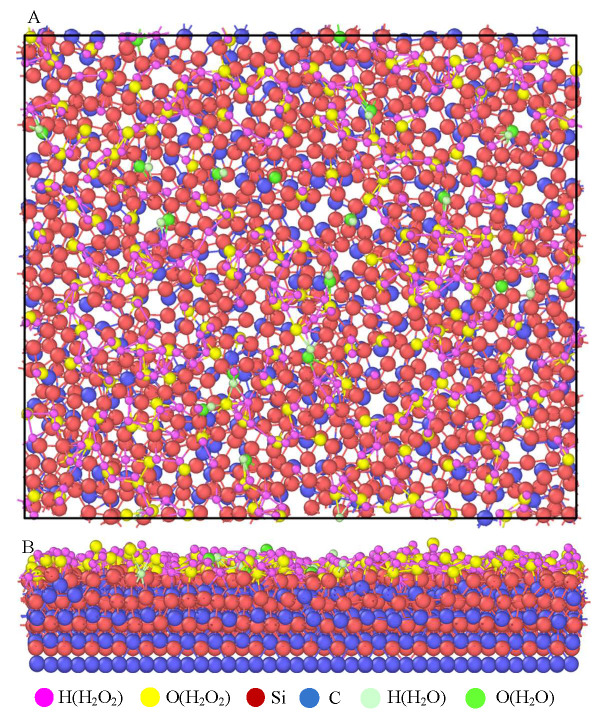
Surface simulation reaction diagram of sodium peroxide polishing solution and the SiC workpiece. (**A**) Top view. (**B**) Frontal view.

**Figure 8:**
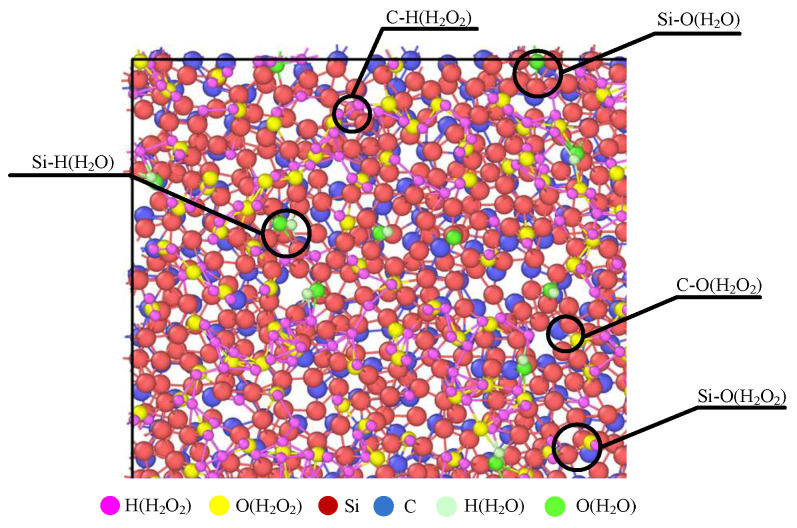
Local analysis of a simulated chemical reaction diagram.

The simulation shows that the polishing liquid will adsorb on the surface of the SiC and form a chemical bond. Figure 7 shows the effect diagram of the simulated chemical reaction process. The ·OH radical in a 30 wt% H_2_O_2_ polishing solution will oxidize the surface of SiC. At the beginning of the reaction, the ·OH radical in the H_2_O_2_ polishing solution will first adsorb on the surface of the workpiece. As the reaction progresses, O atoms and H atoms in H_2_O_2_ solution enter the surface of the SiC workpiece and react with the atoms on the surface of the workpiece to form a new chemical bond, as shown in Figure 8. It will generate Si-O bonds, Si-H bonds, C-O bonds, and C-H bonds. Hence, the reaction simulation indicates that the hydrogen peroxide solution can effectively react with SiC atoms, and even an H_2_O molecule can chemically bond with the Si atom. The simulated results are found to correspond to reference [30], where the Si and C atoms in the system have similar bonding forms of Si-O and C-O.

### 3.2. Subsurface Damage

**Figure 9:**
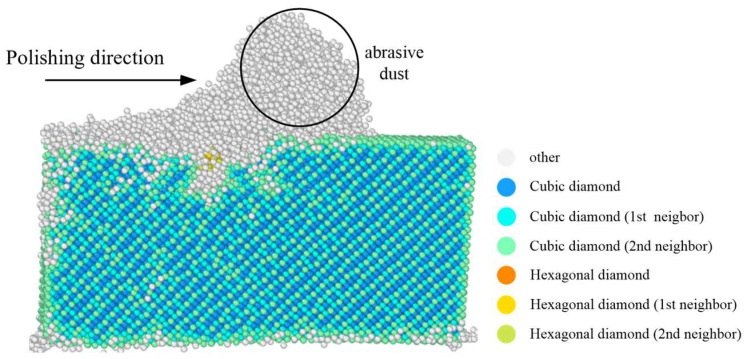
Crystal structure distribution of single-crystal SiC under CMP.

Figure 9 illustrates the atomic-scale structural distribution of SiC following chemical mechanical polishing (CMP), where atoms are color-coded according to their identified crystal structures. The analysis reveals that during the polishing process, the workpiece undergoes significant structural transformations due to mechanical extrusion and sliding interactions with diamond abrasive particles. These transformations result in the formation of a non-original cubic diamond structure, a hexagonal diamond structure, and an amorphous phase. The amorphous structure predominantly forms on the polished surface and within the generated debris, while the subsurface region consists mainly of a reconfigured cubic diamond structure, accompanied by a minor proportion of hexagonal diamond arrangement. These observations suggest that phase transformation and amorphization constitute the primary deformation mechanisms during CMP. Furthermore, although the surface region largely exhibits a cubic diamond structure—as identified via first and second nearest neighbor analysis—this configuration differs from the original crystal structure of SiC. It primarily arises from atomic rearrangement and structural recombination induced by mechanical processing. To further investigate the influence of polishing parameters on the surface integrity of SiC, the surface topography generated under varying processing conditions was systematically analyzed, and the impact of polishing depth and speed was explored.

The atoms of the SiC crystal are colored according to their height in order to better intuitively feel the changes in the surface morphology of the SiC workpiece at different depths and speeds. As shown in Figure 8 and Figure 9, the colored cards in the figure mainly represent the color changes in different heights of SiC at 20~43.5 Å.

**Figure 10:**
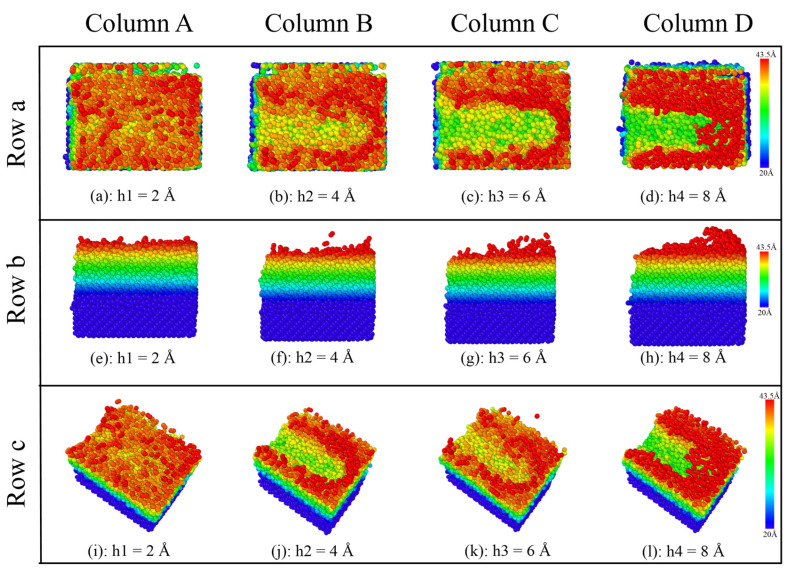
The effect of diamond polishing depth on the surface grinding of the SiC workpiece. (Column A) The depth is 2 Å. (Column B) The depth is 4 Å. (Column C) The depth is 6 Å. (Column D) The depth is 8 Å. (Row a) Front view. (Row b) Right view. (Row c) 3D view.

According to Figure 10, as the sliding depth increases, the groove becomes larger and larger. At a polish depth of 2 Å, the grooves are relatively shallow, and the range of atomic fluctuations on either side is relatively small, even without fluctuation amplitude. As the polish depth increases, the color of the grooves on the surface of the SiC workpiece gradually changes from red to dark green. This means that the friction depth of the SiC workpiece is increasing. When the polishing depth is 8 Å, the groove on the surface of the SiC workpiece is deep, and the range of atomic fluctuations on both sides is also large. At the same time, the accumulation range of the atoms in front of the abrasive particle is also large, as can be seen from the fact that the grooved color of the surface of the SiC workpiece is predominantly dark green.

It can be clearly observed from rows (b) and (c) of Figure 10 that the number of chip atoms produced increases with the increase in depth. In addition, the resulting chip atoms will form a pronounced clustering around the particle-atom front. In the case of a low polishing depth of 2 Å, atoms not only have a low polishing effect on the surface, but also the cross-sectional area does not appear as an accumulation of polishing at a depth of 8 Å. Therefore, the polishing particle must have a certain pressure distance from the depth of the workpiece and thus have a certain effect on the polishing of the workpiece.

**Figure 11:**
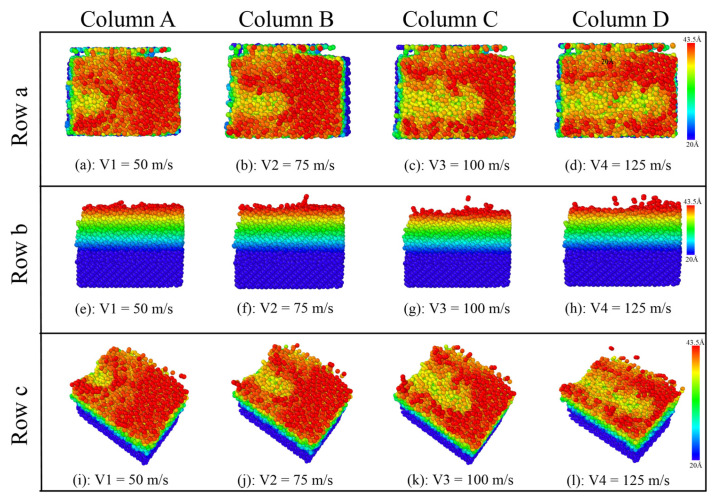
The effect of diamond polishing speed on the surface grinding of the SiC workpiece. (Column A) The speed is 50 m/s. (Column B) The speed is 75 m/s. (Column C) The speed is 100 m/s. (Column D) The speed is 125 m/s. (Row a) Front view. (Row b) Right view. (Row c) 3D view.

As shown in Figure 11, it can be observed that the influence of the diamond polishing speed on the surface polishing effect of the SiC workpiece. As the diamond polishing speed increases, the length of the grooves formed on the surface of the SiC atoms increases significantly, and the amount of atomic motion on the surface increases correspondingly. At a pressure velocity of 50 m/s, the grooves on the surface of the SiC workpiece are relatively shallow, and the range of atomic fluctuations on either side is small or even barely noticeable. The difference in the polishing effect is clearly presented by comparing the polishing velocities of 50 m/s to 100 m/s. As the polishing speed increases, the color of the surface of the SiC workpiece gradually changes from red to dark green, indicating that the friction range of the workpiece surface continues to expand. In particular, when the polishing speed reaches 125 m/s, the groove length on the surface of the SiC workpiece reaches its maximum, covering almost the entire surface of the workpiece, and the fluctuation range of the atoms on both sides is significantly increased.

Based on the data presented in row (b) of Figure 11, it is observed that the interaction area of diamond abrasives expands considerably with increasing polishing speed. At a polishing speed of 50 m/s, the grooves generated by abrasive atoms remain relatively shallow, with limited displacement observed in the surrounding atomic layers. Under these conditions, the predominance of red particles on the SiC workpiece surface indicates a comparatively lower degree of frictional interaction. As the polishing speed increases, a progressive elongation of surface grooves is noted, accompanied by a moderate increase in groove depth. At the maximum tested speed of 125 m/s, substantial groove deepening becomes evident, coupled with significant atomic displacement extending to deeper subsurface layers. These morphological transformations are quantitatively reflected in the expansion of groove cross-sectional area and are further corroborated by distinct colorimetric changes on the workpiece surface.

As observed in row (c) of Figure 11, the diamond polishing speed exhibits no significant correlation with the height of atomic debris accumulated on the workpiece surface. However, an increase in polishing speed is found to induce a moderate rise in the accumulation volume of atoms ahead of the abrasive particles. Furthermore, higher polishing speeds lead to intensified atomic motion within the SiC workpiece. During simulation, distinct particle ejection phenomena can be observed, with certain atoms detaching from the workpiece surface and being propelled into the interfacial region. These collective findings demonstrate that elevated polishing speeds not only influence surface modification but also markedly enhance the dynamic response and atomic-scale material displacement at the workpiece surface.

From the analysis of these experimental phenomena, it can be concluded that increasing the depth and speed of diamond polishing can significantly increase the removal efficiency of SiC surface atoms and thus optimize the processing efficiency. This suggests that an appropriate refinement of the polishing parameters is an effective way to improve the processing efficiency and the quality of the surface.

### 3.3. Atomic Removal Rate

**Figure 12:**
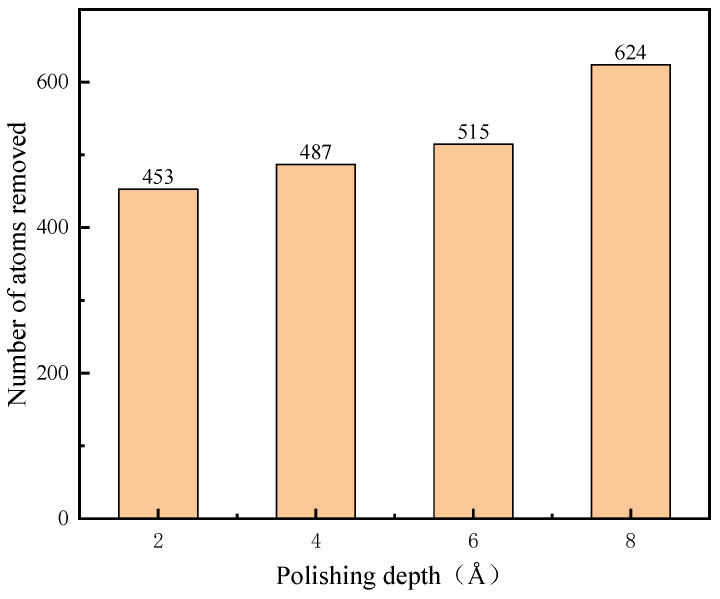
Polishing depth and number of removed atoms.

**Figure 13:**
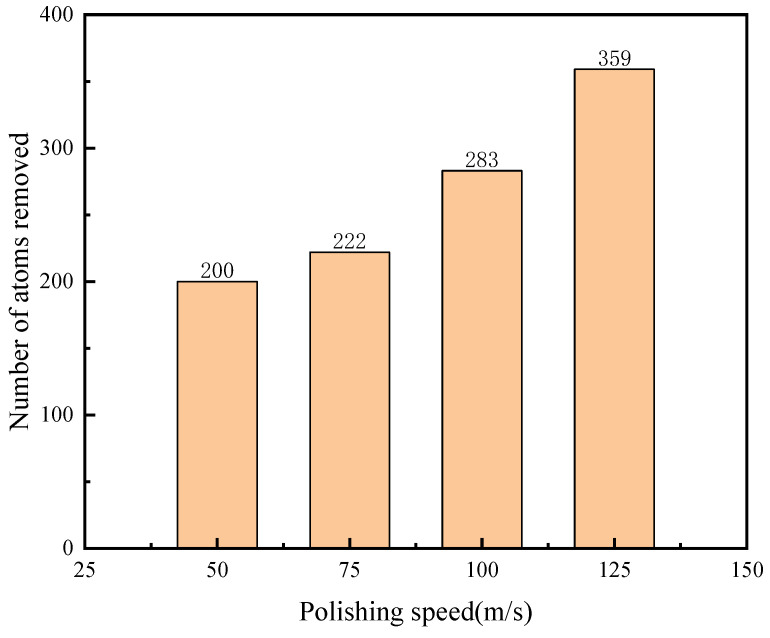
Polishing speed and number of removed atoms.

In this paper, the number of atoms detached from the surface of the workpiece is counted as the removed atoms, that is, the atoms with z coordinates higher than 40 A of the workpiece, so as to analyze the effect of polishing parameters on material removal. The statistics are shown in Figure 12 and Figure 13. The higher the polishing depth and polishing speed, the higher the atom removal rate, especially as the change in the polishing speed makes the change in the atom removal number very obvious.

**Figure 14:**
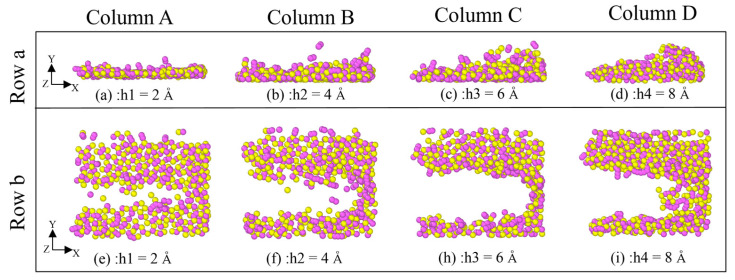
The effect of diamond polishing depth on the removal of atoms on the surface of the SiC workpiece. (Column A) The depth is 2 Å. (Column B) The depth is 4 Å. (Column C) The depth is 6 Å. (Column D) The depth is 8 Å. (Row a) Right view. (Row b) Top view.

**Figure 15:**
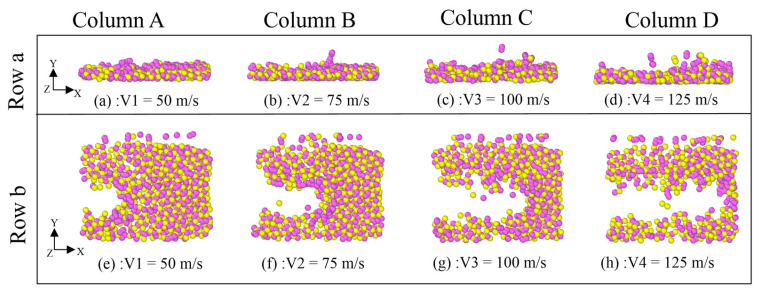
Effect of diamond polishing speed on the removal of atoms from the surface of the SiC workpiece. (Column A) The speed is 50 m/s. (Column B) The speed is 75 m/s. (Column C) The speed is 100 m/s. (Column D) The speed is 125 m/s. (Row a) Right view. (Row b) Top view.

As shown in the top-view and front-view renderings of workpiece regions with z-coordinates above 40 Å (Figure 14a and Figure 15a), the workpiece atoms undergo significant extrusion and shear deformation under the action of abrasive particles. A high-density arc-shaped chip accumulation forms ahead of the abrasive tip, while no substantial lateral chip flow is observed along the sides of the diamond particle. Instead, the lattice surface exhibits irregular extrusion and deformation with varying degrees of severity. According to the results presented in row (b) of Figure 14 and Figure 15, both the number of dislodged atoms and the accumulation height increase progressively with greater polishing depth and higher polishing speeds. As the abrasive particle advances, it continuously displaces surrounding SiC atoms, leading to the formation of taller accumulations and a corresponding expansion in the occupied volume. This behavior can be attributed to the deeper penetration of the abrasive into the workpiece, which intensifies mutual extrusion between the diamond particle and the SiC substrate, thereby elevating the chip accumulation height. These observed mechanisms contribute not only to enhanced material removal efficiency but also to an overall improvement in process efficiency during polishing. Based on the above-mentioned simulation, it can be seen that pressure may have a significant impact on the polishing performance. High polishing can achieve high material removal quantity, which corresponds to the findings in reference [31].

### 3.4. Polishing Force and Temperature

#### 3.4.1. Polishing Force

In MD simulations, the polishing force depends on the interatomic interaction. The magnitude of the polishing force not only affects the deformation of the work surface but also determines the accuracy and quality of the workpiece. When abrasive particles come into contact with the workpiece, the workpiece undergoes elastic and plastic deformations. The friction generated by overcoming the elastic and plastic deformations of the workpiece is the main source of polishing force. The change in the polishing force has a crucial effect on the removal of atoms from the surface of the material. The polishing force can be changed by changing the polishing parameters.

**Figure 16:**
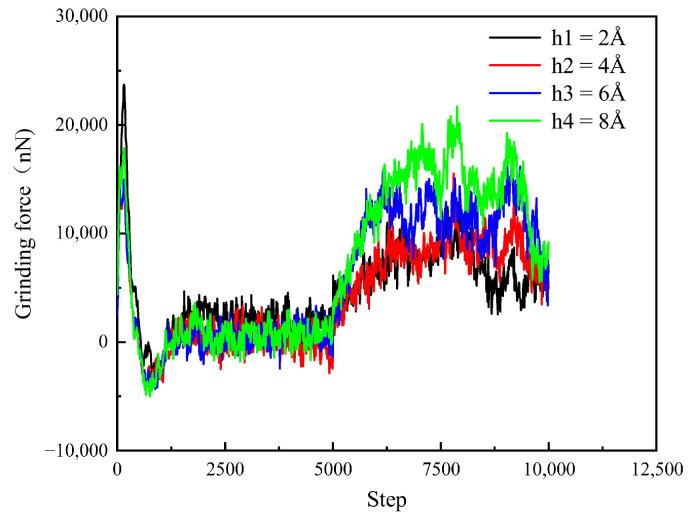
Change in polishing force of SiC at different polishing depths.

As can be seen in Figure 16, before the relaxation, not only does the polishing force on the workpiece fluctuate up and down in the same region, but the amplitude of the fluctuations between different depths is almost the same. After relaxation, the diamond officially began the polishing process. At the beginning of the polishing process, the number of atoms in contact between the diamond particles and the surface of the SiC workpiece is small, and the polishing force is low. However, the polishing force increases with the step size. The deeper the pressure, the greater the polishing pressure. The deeper the polishing depth, the larger the actual area and number of atoms of the diamond in contact with the workpiece, thus making the force between them more obvious.

**Figure 17:**
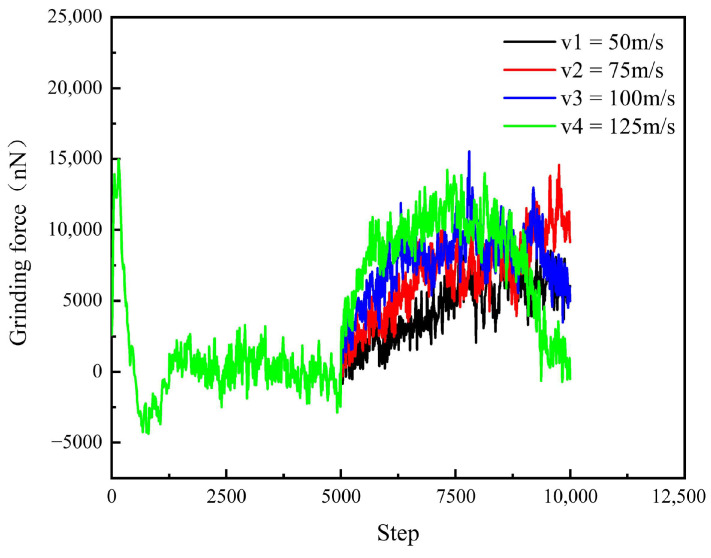
Change in polishing force of SiC at different polishing speeds.

As can be seen from Figure 17, in the simulation relaxation process, although the polishing speed of the abrasive particles is different, the polishing force of SiC remains constant or even has no obvious difference. The reason for the consistent polishing force during this time is that the abrasive particles have not yet made contact with the workpiece surface, and the polishing operations are carried out at the same depth. At the beginning of polishing, the number of atoms in contact with the substrate is small. With the polishing process, the reason that the polishing force will gradually increase is that the chemical bond between the atoms inside the SiC workpiece will be broken and accompanied by the generation of new bonds, resulting in crystal defects. At the same time, when the polishing speed increases, the number of atoms in contact with the diamond and the workpiece will also increase, making the interaction between the two more significant, and thus the polishing pressure will also increase.

#### 3.4.2. Temperature

In the simulation experiment of CMP, the surface temperature of SiC not only affects the deformation of the workpiece surface but also indirectly affects the atomic movement. Temperature changes are critical to the removal of atoms from the surface of a material. The change in polishing parameters can change the surface temperature of the workpiece.

**Figure 18:**
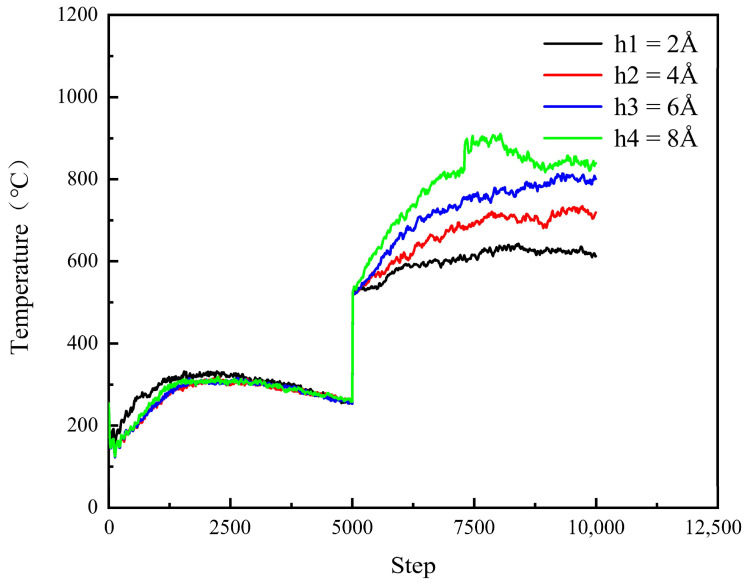
Surface temperature variation in the SiC workpiece at different polishing depths.

As illustrated in Figure 18, the surface temperature of the workpiece exhibits minor fluctuations around a stable baseline prior to the relaxation phase. Following the onset of polishing, however, the surface temperature of the SiC substrate demonstrates significant variation, strongly correlated with the applied polishing depth. This thermal response is attributed to the penetration of the diamond abrasive into the workpiece, which induces frictional heating and plastic deformation at the contact interface. Subsequently, a marked increase in workpiece surface temperature is observed, with deeper indentation depths resulting in higher peak temperatures. Two primary mechanisms underlie this phenomenon. First, greater indentation depth amplifies the number of displaced atoms and intensifies interatomic interactions, thereby elevating the intrinsic energy dissipation. Second, the enlarged contact area between the abrasive and the workpiece facilitates enhanced thermal energy transfer into the substrate, further contributing to the temperature rise.

**Figure 19:**
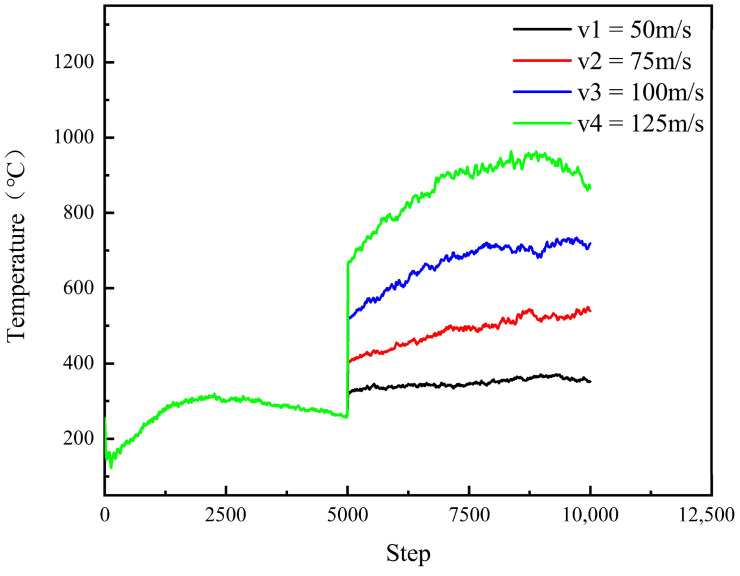
Surface temperature variation in the SiC workpiece at different polishing speeds.

As shown in Figure 19, during the relaxation phase, both the surface temperature and polishing force of the SiC workpiece remain stable across different abrasive particle speeds. Following relaxation, however, the surface temperature exhibits a pronounced dependence on polishing speed, a response attributable to frictional and extrusion interactions induced by diamond abrasives on the workpiece surface. Subsequently, the workpiece temperature demonstrates a continuous increase. When the polishing speed is raised from 50 m/s to 125 m/s, although the heating rates vary, the maximum temperature rises in an approximately linear manner. This trend can be explained by the accelerated heat accumulation at higher speeds, where the generated thermal energy cannot be dissipated sufficiently during the process, leading to a progressive increase in polishing temperature. In general, the temperature of the workpiece rises significantly during polishing due to mechanical extrusion and shear deformation caused by the abrasive particles. A portion of the mechanical energy input is converted into thermal energy, thereby increasing the kinetic energy of workpiece atoms.

### 3.5. Reaxff-Based Polishing Simulation

**Figure 20:**
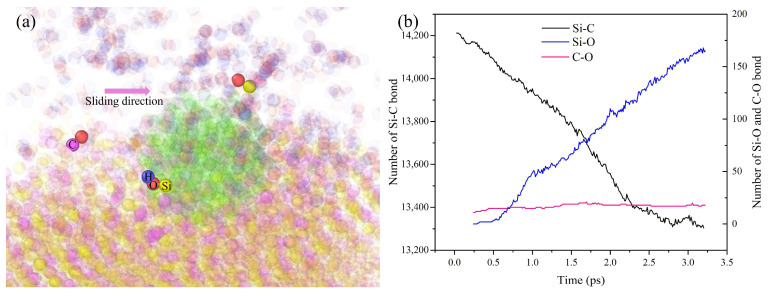
Simulation under the Reaxff force field: (**a**) atom removal form, (**b**) bond number variation during simulation.

To further investigate the atom removal mechanism of CMP under the reaction of hydrogen peroxide, the Reaxff-based polishing simulation is conducted. The specific simulation parameters are shown in Table 4. Figure 20a shows the polishing process under the Reaxff force field. It can be shown that the Si atoms are removed in the form of Si-O bonds, while the C atoms are mainly in the form of a C-O bond, which accords with the reaction behavior in the oxidation simulation. Figure 20b shows different bond type variations during simulation. It can be seen that the Si-C bond keeps decreasing during simulation, while the Si-O and C-O show an increasing trend. This indicates that during the simulation, there are Si or C atoms that are removed through the mechanical action or the chemical reaction product. 

## 4. Conclusions

The effect of different process parameters on the CMP effect of SiC was deeply investigated by simulation, and the deep mechanism of material removal was revealed by analyzing the surface morphology of SiC. Through this series of studies, the following important conclusions can be drawn:

1. In the oxidation simulation, the Si and C atoms are oxidized in different forms. The Si atoms are in the form of Si-O, Si-H, and Si-H_2_O, while the C atom is mainly in form of C-O. In addition, simulation results show that reaction is not only caused by the H_2_O_2_, H_2_O molecule can also chemically absorb on the SiC surface.
2. With the increase in diamond polishing depth, the grooves on the surface of the SiC workpiece are gradually deeper, and the range of atomic fluctuations on both sides is also increasing. When the polishing depth reached 8 angstroms, the highest polishing force reached around 15000 nN, and the instantaneous temperature reached around 800 °C. After polishing, the removed atoms reached 624.
3. As the speed of diamond polishing continues to increase, the area of diamond movement is becoming larger. Polishing speed mainly affects the polishing length at a set time duration. When the polishing speed is high, the abrasive can slide through a longer distance, thus producing more atom removal. Hence, when the polishing speed reached 125 m/s, the atom removal number reached 359, and the maximum polishing force reached 1000 nN. In addition, the higher the polishing speed, the higher the instantaneous kinetic energy. In this case, when the abrasive meets the substrate, the higher polishing force can be observed.
4. Exploration of polishing speed and polishing depth on the polishing performance reveals that the polishing depth has a more significant impact. Compared to the polishing speed, when the polishing depth increases, a higher polishing force can be generated. This indicates that the polishing pressure has an important influence on the polishing process of the SiC wafer.
5. Limitations and future works: This work explores the potential of H_2_O_2_ solution in the 4H-SiC wafer polishing. However, the fundamental mechanism of the oxidation behavior of the H_2_O_2_ on the 4H-SiC remained unexplored. The first-principle calculation can analyze the chemical behavior through the wave function generated during the calculation. The wave function contains all the chemical information (e.g., electron density, covalent bond, atom charge…). For future work, the first-principles method can be employed to further analyze the chemical behaviors during CMP.

## Figures and Tables

**Table 1:** Simulation parameters of a chemical reaction.

Parameter	Settings
Workpiece material	Single-crystal 4H-SiC
Dimension of the workpiece	75 Å × 54 Å × 25 Å
Number of workpiece atoms	3840
Polishing solution	30 wt%H_2_O_2_
Number of atoms in polishing solution	44
Potential energy function	Tersoff
Boundary condition	P P P
Ensemble	NVE
Initial temperature	300 K

**Table 2:** Simulation parameters of chemical mechanical polishing of SiC.

Parameter	Settings
Workpiece material	Single-crystal 4H-SiC
Dimension of the workpiece	55 Å × 33 Å × 40 Å
Number of workpiece atoms	8480
Abrasive material	Diamond
Abrasive radius	8 Å
Number of abrasive atoms	372
Scratching depth	2, 4, 6, and 8 Å
Scratching velocity	50, 75, 100, 125 m/s
Potential energy function	Tersoff, LJ
Boundary condition	P P P
Ensemble	NVE
Initial temperature	300 K

**Table 3:** Interatomic parameters of atoms from different groups (LJ potential).

Atom Pair	Epsilon (eV)	Sigma (Angstrom)
Si-C_diamond_	0.0085	4.067
Si-O	0.0061	3.693
C-O	0.0121	3.890

**Table 4:** Simulation parameters of the Reaxff force field.

Parameter	Settings
Workpiece material	Single-crystal 4H-SiC
Dimension of the workpiece	55 Å × 33 Å × 40 Å
Number of workpiece atoms	8480
Abrasive material	Diamond
Abrasive radius	8 Å
Number of abrasive atoms	372
Potential energy function	Reaxff [32]
Boundary condition	P P P
Ensemble	NVE
Initial temperature	300 K

## Data Availability

The original contributions presented in this study are included in the article. Further inquiries can be directed to the corresponding author.

## References

[1] Wang X. (2022). Application status and development trend of semiconductor materials. Lamps Light..

[2] Tang A.L. (2023). Study on Mechanism of Vibration-Assisted Photocatalytic Polishing of Silicon Carbide. Master’s Thesis.

[3] Chen Y. (2023). Research on the Mechanism of Axial Ultrasonic Vibration Assisted Scraping of Single Crystal Silicon Carbide Based on Molecular Dynamics Simulation. Ph.D. Thesis.

[4] Gao B., Zhai W.J., Zhai Q., Wang C. (2021). Novel photoelectrochemically combined mechanical polishing technology for scratch-free 4H-SiC surface by using CeO_2_-TiO_2_ composite photocatalysts and PS/CeO_2_ core/shell abrasives. Appl. Surf. Sci..

[5] Huang S.Q., Gao S., Huang C.Z., Huang H. (2022). Nanoscale removal mechanisms in abrasive machining of brittle solids. Diam. Abras. Eng..

[6] Luo Q.F., Lu J., Tian Z.G., Jiang F. (2021). Controllable material removal behavior of 6H-SiC wafer in nanoscale polishing. Appl. Surf. Sci..

[7] Meng B.B., Yuan D.D., Zheng J., Qiu P., Xu S.L. (2020). Tip-based nanomanufacturing process of single crystal SiC: Ductile deformation mechanism and process optimization. Appl. Surf. Sci..

[8] Wei S.S., Yin Z.P., Bai J., Xie W.W., Qin F.W., Su Y., Wang D.J. (2022). The structural and electronic properties of Carbon-related point defects on 4H-SiC (0001) surface. Appl. Surf. Sci..

[9] Shi Z.Y. (2020). Study on CMP Method and Material Removal Mechanism of Single Crystal Diamond at Room Temperature. Ph.D. Thesis.

[10] Zhou L., Audurier V., Pirouz P., Powell J.A. (1997). Chemomechanical polishing of silicon carbide. J. Electrochem. Soc..

[11] Sun X.H., Li J.H., Zhang W., Zeng Q.F., Zhang J.F. (2024). Research progress of material removal uniformity in chemical-mechanical polishing of silicon carbide. J. Synth. Cryst..

[12] Lee H., Park B., Lee H.S., Jeong S., Seo H.D., Joo S., Jeong H., Kim H.J. (2009). The effect of mixed abrasive slurry on CMP of 6H-SiC substrates. J. Ceram. Process. Res..

[13] Chen G.M. (2017). Study on Ultra-Precision Polishing Technology and Mechanism of Silicon Carbide Wafer. Ph.D. Thesis.

[14] Pang L.F., Li X.B., Li T.T., Jia J.P. (2021). Ultra precision CMP technology for SiC wafer. Micronanoelectron. Technol..

[15] Xue M.P., Xiao W., Zhang T.Y., Wang Z.K., Su J.X. (2023). Catalytic mechanism of triboCMP on (0001) C-face of single crystal 6H-SiC substrate. Int. J. Adv. Manuf. Technol..

[16] Zhai W.J., Yang D.C., Gong N. (2018). Molecular dynamics simulation of polishing process of silicon carbide under ultrasonic vibration. J. Shanghai Jiaotong Univ..

[17] Wang G.L., Zhang G.H., Wang Z.G., Feng Z.J. (2021). Molecular dynamics simulation of the effect of pressure on phase transformation of nano-polished silicon carbide. Mach. Des. Manuf..

[18] Sun Q., Xu J.X., Lu K., Chu Z.H. (2021). Microsimulation analysis of the interaction between diamond abrasive and silica abrasive in chemical-mechanical polishing of SiC. Electroplat. Finish..

[19] Tang A.L., Fan Z.W., Tang M.L., Wang Y. (2024). Effect of abrasive particle vibration on microstructure evolution and material removal of silicon carbide CMP. Diam. Abras. Eng..

[20] Wang M., Duan F. (2021). Atomic-level material removal mechanisms of Si (110) chemical mechanical polishing: Insights from ReaxFF reactive molecular dynamics simulations. Langmuir.

[21] Ban X.X., Zhu J.H., Sun G.N., Han S.X., Duan T.X., Wang N.C. (2024). Molecular simulation of ultrasonic assisted diamond grit scratching 4H-SiC single-crystal. Tribol. Int..

[22] Gao S., Wang H., Huang H., Kang R.K. (2023). Molecular simulation of the plastic deformation and crack formation in single grit polishing of 4H-SiC single crystal. Int. J. Mech. Sci..

[23] He Y., Yuan Z., Tang M., Sun J., Liu C., Gao X. (2022). Mechanism of chemical and mechanical mutual promotion in photocatalysis-assisted CMP for single-crystal SiC. Proc. Inst. Mech. Eng. Part C J. Mech. Eng. Sci..

[24] Wang W., Lu X., Wu X., Wang R., Deren Y., Pi X. (2025). Oxidation anisotropy of 4H-SiC wafers during chemical-mechanical polishing. Mater. Sci. Semicond. Process..

[25] Kang H., Zhong M., Li X., Yi M., Chen J., Xu W. (2025). Investigation of Fenton-electrochemical oxidation behavior and polishing mechanism of SiC. Precis. Eng..

[26] He L., Li J., Tang C., Chen K., Yang L., Si J. (2025). ReaxFF molecular dynamics of chemical reaction mechanism of SiC crystal with alcoholic additives in CMP. Comput. Mater. Sci..

[27] Cui D., Zhang B., Xian W., Liu M., Liu S., Wu P., Wang Y. (2025). Comparative study on MnO_2_, Mn_2_O_3_, and Mn_3_O_4_: Enhancing chemical-mechanical polishing properties of 4H-SiC silicon wafers. Mater. Sci. Semicond. Process..

[28] Zhao Y., Gao S., Sun Y., Wu Y., Yang F., Kang R., Dong Z. (2025). Electric-field-modulated oxidation and its effect on photoelectrochemical mechanical polishing of 4H-SiC. Int. J. Mech. Sci..

[29] Han Z., Ran B., Pan J., Zhuang R. (2025). Investigation of the Visible Photocatalytic–Fenton Reactive Composite Polishing Process for Single-Crystal SiC Wafers Based on Response Surface Methodology. Micromachines.

[30] Zhou Y., Xu K., Gao Y., Yu Z., Zhu F. (2024). Effects of oxidizer concentration and abrasive type on interfacial bonding and material removal in 4H-SiC polishing processes. Phys. Chem. Chem. Phys..

[31] Wu P., Liu N., Li X., Zhu Y. (2024). Material removal rate model for chemical–mechanical polishing of single-crystal SiC substrates using agglomerated diamond abrasive. Precis. Eng..

[32] Soria F.A., Zhang W., Paredes-Olivera P.A., van Duin A.C.T., Patrito E.M. (2018). Si/C/H ReaxFF Reactive Potential for Silicon Surfaces Grafted with Organic Molecules. J. Phys. Chem. C.

